# Using Slack to communicate with medical students

**DOI:** 10.5195/jmla.2018.482

**Published:** 2018-10-01

**Authors:** Kathryn Vela

**Affiliations:** Health Sciences Outreach Librarian, Spokane Academic Library. Elson S. Floyd College of Medicine, Washington State University, Spokane, WA

## Abstract

Academic libraries often make use of social networks like Facebook and Twitter to connect with their student users. While the Spokane Academic Library at Washington State University also employs this outreach strategy, the health sciences librarian was able to use a new type of social network called Slack to communicate more directly with the inaugural class of the Elson S. Floyd College of Medicine. As a digital workspace for communication and collaboration, Slack provided the medical librarian with an effective medium through which to post library announcements and updates, as well as have individual conversations with students about library-related questions and concerns.

## INTRODUCTION

Washington State University (WSU) is a land-grant institution located in Pullman, Washington, on the eastern side of the state. In addition to this main campus, there are also campuses in Everett, Spokane, Tri-Cities, and Vancouver. The Spokane campus is home to most of WSU’s health sciences degrees, which historically have included nursing, pharmacy, speech and hearing sciences, and nutrition and exercise physiology. In 2015, a new medical school was founded at WSU to address the persistent shortage of health care professionals in Washington State, particularly in the rural eastern side of the state [1]. The Elson S. Floyd College of Medicine (ESFCOM) accepted its first class of sixty students in August 2017.

The Spokane campus is served by the Spokane Academic Library (SAL), a joint-use library that is shared with Eastern Washington University. I was hired as the health sciences librarian for ESFCOM in August 2016, one year prior to the start of the curriculum. While I was able to establish regular contact with faculty through membership on the curriculum committee, I was unable to integrate myself into the curriculum itself beyond a two-hour session during the two-week orientation period and a small number of brief journal club presentations. As Pozdol said, “students who get to know librarians on a more personal level are more likely to feel comfortable asking for help” [2]. Therefore, I needed to find a way to have more regular contact with the medical students to build a trusting and productive relationship with them.

SAL primarily communicates with students through a variety of methods, including campus email announcements, digital signage, and a library whiteboard. SAL also has a Facebook page with 277 followers and a Twitter account with 396 followers. Both accounts are fairly active, with frequent posts about workshops, library hours, and online resources. While these methods are suitable for general library announcements, it can be more challenging to meaningfully engage with students on these platforms.

## TRENDS IN SOCIAL NETWORKING PLATFORMS

The use of social networks in academic libraries to communicate with students and faculty is a common practice. The Library 2.0 movement describes the efforts of libraries and librarians to adopt new technologies to engage with users online, and social networks are one of the most frequently used technologies to achieve this goal [3]. The traditional role of libraries and librarians is to “provide both physical and virtual spaces for community, helping them grow, collaborate, and communicate” [4], so it is no surprise that academic libraries have so readily adopted social networking platforms as a means to connect with their users.

King describes five primary reasons for libraries to use social networking platforms: (1) listening to user needs and interests, (2) making online connections with users, (3) soliciting thoughts and opinions from users, (4) using the convenience of mobile technology, and (5) having the ability to reach more users where they are [5]. Many academic libraries have realized these benefits, with 49% of academic research libraries using Facebook and 46% using Twitter [6]. Yet, libraries have faced challenges in taking advantage of these social networking platforms as well: Chu and Du report that library staff often do not have the time to effectively manage their library’s social media channels, lack understanding of how to use the social media channels, and struggle with determining how exactly to communicate with their user groups [7].

## SLACK AS A POTENTIAL COMMUNICATION CHANNEL

One relatively recent addition to the suite of social networking platforms is Slack, a digital workspace that allows users to collaborate on group projects in teams. Slack is part of a group of similar platforms known as “enterprise social networks” (ESNs), which are specifically designed for organizations to use [4]. While Facebook and Twitter provide users with the means to create and promote a personal image, Slack and other similar platforms aim to allow users to share expertise and support collaboration [8]. Platforms like Slack offer a more streamlined method of communication than platforms like Facebook and Twitter, creating a richer knowledge-sharing environment [8].

Users can create multiple channels in a Slack workspace to house messages, files, and links related to different topics or projects. Members of a team can communicate with the entire group using public messages in a channel or communicate directly with a specific person with a direct message. A key feature of Slack is its searchability: all messages and files posted in Slack are searchable. Users can also choose to receive notifications of new content via desktop, mobile device, or email. It is available on Mac, Windows, and Linux, in both desktop and app versions. For small groups or those planning to use the platform temporarily, Slack is free of charge, with some limits to usage and functionality. For larger groups or groups with more complex collaboration needs, Slack offers per-user monthly pricing for expanded access to storage and features [9, 10]. ESFCOM subscribes to the standard plan, which provides enough functionality for the college to use it as a central communication hub.

Slack has the potential to provide librarians with a new medium through which to communicate with their user bases. Rather than relying on promotional strategies and complex interfaces, Slack is modeled on the familiar instant-messaging structure that emerged during the earliest years of the Internet. It allows an easier and more natural back-and-forth interaction, similar to what is experienced during a traditional reference interaction. Even so, Slack includes more advanced capabilities for those who feel comfortable using them, like loading files and images, tagging messages, and creating customized add-ons.

## SLACK AT THE ELSON S. FLOYD COLLEGE OF MEDICINE

Prior to the start of their first term, the inaugural class of ESFCOM medical students created a group on Slack to communicate and collaborate with each other. Since they began using Slack, seventy different channels have been created in the ESFCOM workspace. Some channels are formed by students to connect and collaborate with their peers, while others are formed by instructors to recruit and organize students for projects of interest. While most topics are related to the medical curriculum, like #emergency_interest and #familymed, others are more extracurricular in nature, such as #running and #wsu_climbing. There are also the Slack default channels #general, for program-wide announcements, and #random, for “non-work banter and watercooler conversation.”

SAL staff created a channel in the ESFCOM workspace with the assistance of ESFCOM information technology (IT) in August 2017, not long after the first cohort of medical students began attending classes. The channel is called #wsu-spokane-library, and I use it in a variety of ways to stay connected to the medical students. It provides the ideal medium for new resource announcements, updates to the Medicine Research Guide, and reminders of existing library services and resources. In addition, I can provide information about network issues and work with students to troubleshoot connectivity issues that they may be having with library resources both on- and off-campus.

The direct message (DM) feature of Slack has proved to be a valuable tool in communicating with individual medical students about a wide range of topics. There have been concerns about library noise and study room availability that were resolved through DM conversations, as well as brief reference questions and requests for consultation appointments. I also collaborate with ESFCOM IT through their #IT channel to resolve issues about access to library resources for individual students.

This has been particularly useful during the early part of the 2017 academic year, as the library experienced challenges in off-campus access with some newly acquired medical databases. As the library worked with the vendors in question to remedy the problem and establish consistent network connections, I was able to use Slack to communicate with students who were experiencing off-campus access issues as a result of the problem. Students appreciated the prompt responses to their questions about off-campus access and notifications about when a solution would be in place.

## BENEFITS AND DRAWBACKS TO SLACK AS A COMMUNICATION CHANNEL

A number of benefits to using Slack as a library communication channel emerged during my use of Slack with the medical students at ESFCOM. The convenience of the platform makes it easy for libraries to post announcements and hold conversations with students in real time, rather than waiting up to a day for communications to be distributed through the campus-wide email discussion list. It also allows common library issues like network access to be resolved at the time of need, which can be critical when many academic curricula rely on resources that are electronically available through the library.

A library Slack channel can also function as a medium through which students can ask questions of the librarians for a quick response. With busy schedules, many students are not left with much spare time that they can use to meet with a librarian for reference consultations. And in the case of SAL, there is no other instant-messaging chat service for brief reference questions. Slack allows students to send questions directly to a librarian through the DM feature or through the library channel itself.

While the convenience and ease of communication afforded by Slack can be beneficial, there can also be challenges with attracting and keeping the attention of students who have not sought assistance with library resources. For instance, when the #wsu-spokane-library channel was first created, every student in the first ESFCOM cohort was added to the channel, for a total of sixty student participants. Over the first few months of the curriculum, approximately six people left the channel for unknown reasons. This means that a small percentage of medical students are not receiving the library announcements and updates through Slack and might not be taking advantage of the information services that the library provides.

A downside to using Slack is the difficulty in accessing or collecting data to evaluate the platform and the library channel. The channel owner has access to a small number of basic statistics, such as the total number of people in the channel and the message “reactions” that people can choose to post. Beyond these scant data, there is not a means to quickly determine how people are interacting on the library channel. Due to this shortcoming, future research could involve a formal assessment of the #wsu-spokane-library Slack channel in order to get a sense of the success of the channel in communicating with the medical students.

## CONCLUSION

Finding a reliable way to connect with their student base is a challenging task for any librarian. The best and most effective method will vary depending on each student population, so it is important that librarians keep an open mind and are willing to try new communication techniques and technologies. The use of Slack, as described here, is just one example of how librarians can take advantage of new methods of communication in order to establish a positive presence in students’ academic lives.
